# A Positive Feedback Loop between Inactive VHL-Triggered Histone Lactylation and PDGFRβ Signaling Drives Clear Cell Renal Cell Carcinoma Progression

**DOI:** 10.7150/ijbs.73398

**Published:** 2022-05-13

**Authors:** Jiefeng Yang, Li Luo, Chongyu Zhao, Xiyuan Li, Zimo Wang, Ziwei Zeng, Xin Yang, Xiaobin Zheng, Haiqing Jie, Liang Kang, Shujuan Li, Shuang Liu, Chi Zhou, Huashan Liu

**Affiliations:** 1 State Key Laboratory of Oncology in South China, Collaborative Innovation Center for Cancer Medicine, Sun Yat-sen University Cancer Center, Guangzhou, Guangdong, China; 2 Department of Colorectal Surgery and Guangdong Provincial Key Laboratory of Colorectal and Pelvic Floor Diseases, The Sixth Affiliated Hospital, Sun Yat-sen University, Guangzhou, Guangdong, China; 3 Department of Urology, The First Affiliated Hospital, Sun Yat-sen University, Guangzhou, Guangdong, China; 4 Department of Colorectal Surgery, Sun Yat-sen University Cancer Center, Guangzhou, Guangdong, China; 5 Department of Hepatobiliary Oncology, Sun Yat-sen University Cancer Center, Guangzhou, Guangdong, China; 6 Department of Pancreatobiliary Surgery, Sun Yat-sen University Cancer Center, Guangzhou, Guangdong, China; 7 Department of Head and Neck Surgery, Sun Yat-sen University Cancer Center, Guangzhou, Guangdong, China; 8 Department of Ultrasound, Second Clinical College of Jinan University, Shenzhen People's Hospital, Shenzhen, Guangdong, China; 9 Department of Pharmacy, The Third Affiliated Hospital of Zhengzhou University, Zhengzhou, Henan, China

**Keywords:** Histone lactylation, inactive VHL, PDGFRβ, ccRCC

## Abstract

Inactive von Hippel-Lindau (VHL) is linked to metabolic reprogramming and plays pivotal roles in the pathogenesis of clear cell renal cell carcinoma (ccRCC). Here, we identify a previously unknown oncogenic role for inactive VHL in actively triggering histone lactylation to promote ccRCC progression. In patients with ccRCC, inactive VHL positively correlates with the presence of histone lactylation, and high levels of histone lactylation indicates poor patient prognosis. Inactive VHL-triggered histone lactylation contributes to ccRCC progression by activating the transcription of platelet-derived growth factor receptor β (PDGFRβ). In turn, PDGFRβ signaling is shown to stimulate histone lactylation, thereby forming an oncogenic positive feedback loop in ccRCC. Target correction of aberrant histone lactylation represses the growth and metastasis of ccRCC in vivo. More importantly, the combined inhibition of histone lactylation and PDGFRβ significantly reinforces the therapeutic efficacy. This work underscores the importance of histone lactylation in facilitating ccRCC progression and suggests targeting the positive feedback loop between histone lactylation and PDGFRβ signaling might provide a promising therapeutic strategy for ccRCC patients.

## Introduction

As one of the most common malignant tumors, clear cell renal cell carcinoma (ccRCC) accounts for 70-80% of all kidney cancers and remains a leading cause of cancer-related morbidity and mortality worldwide 1-3. The current therapeutic strategy has offered a substantial improvement in clinical outcomes of patients with localized ccRCC 4. Unfortunately, it remains a daunting challenge of the present therapies for patients with ccRCC at advanced stages, leaving 40% of those having distant metastasis and a 5-year survival rate of less than 10% in patients with metastatic ccRCC 5-7. These insights indicate an unmet need for alternative treatment options for ccRCC.

The von Hippel-Lindau (VHL) mutation, which is widely recognized as an essential contributor for the pathogenesis of ccRCC, was reported to arise in up to 90% of patients with ccRCC 2, 8. The functions of VHL mutation on ccRCC pathogenesis were faithfully indicated by genetically engineered ccRCC mouse models, where VHL loss plays vital roles in the formation and progression of ccRCC 9, 10. Moreover, several clinically approved agents targeting VHL inactivation-associated downstream pathways (for example, Sunitinib, Axitinib, and Sorafenib) have confirmed efficacy in treating ccRCC 11. These facets suggest VHL performs complex roles in ccRCC. Further understanding the molecular mechanisms of VHL mutation could be helpful to facilitate the development of better therapeutic strategies to improve clinical outcomes.

Currently, VHL loss in ccRCC results in multiple effects, the best-documented of which correlates with its capacity of targeting hypoxia-inducible factors (HIFs) 11, 12. Given the well-established link of VHL/HIF pathway with glycolysis-derived lactate 13, high levels of lactate represent a major hallmark of ccRCC 14. Since its first description in 2019 15, histone lactylation has been seen in multiple diseases 16, 17, and has also been identified as a crucial orchestrator for the tumorigenesis by driving oncogenic gene expression 18. However, the role of histone lactylation remains an important knowledge gap in our understanding of ccRCC pathogenesis. Therefore, this work set out to gain insights into the contribution of histone lactylation to ccRCC and the mechanism by which this arises.

## Materials and methods

Detailed procedures are provided in Supplementary materials.

### Patients and tissue samples

Formalin-fixed, paraffin-embedded ccRCC tissues were obtained from 65 patients who had undergone operation at the Sun Yat-sen University Cancer Center. All related procedures involving human samples were approved by the Institutional Ethical Review Boards of the Sun Yat-sen University Cancer Center, and informed written consent was obtained from all subjects.

### Cell culture

Human immortalized renal epithelial cell HK2, and human RCC cell lines 786-O, A498, Caki-1 and ACHN were from the Chinese Academy of Science. Cells were cultured at 37 °C in a humidified incubator with 5% CO_2_, in DMEM medium containing 10% FBS (HK2, A498 and ACHN), RPMI-1640 medium supplemented with 10% FBS (786-O) and McCoy's 5 A medium with 10% FBS (Caki-1).

### In vivo tumor xenograft models

Animal experiments were approved by the Institutional Animal Care and Use Committee of the Sun Yat-sen University and conformed to the “Guide for the Care and Use of Laboratory Animals” of the National Institute of Health in China. BALB/c nude mice and NSG mice were maintained in pathogen free conditions at the Experimental Animal Center of Sun Yat-sen University.

For patient derived xenograft (PDX) mice models, freshly sectioned ccRCC tissues were transported in RPMI 1640 on ice and minced finely into fragments of 1-2 mm^3^, mixed with Matrigel, and implanted subcutaneously into the right flank of 6-week-old male immunocompromised NSG mice. When the tumors reached volumes of about 100 mm^3^, the mice were randomly assigned to the following 4 treatments (n =5 per group): vehicle, oxamate (60 mg/kg, daily for six weeks), Axitinib (30 mg/kg/d, daily for four weeks with a two-week interval), or both oxamate and Axitinib. Tumor size was then measured every week, and was estimated using the following formula: Volume = (Longer diameter × Shorter diameter^2^)/2. The mice were sacrificed and the weight of the tumor was measured at 6 weeks after the indicated treatment.

For in vivo metastasis studies, 5×10^5^ 786-O cells were injected into the tail veins of 6-week-old BALB/c nude mice. Each group consisted of 5 animals. After 5 weeks, the mice were sacrificed and the lungs were harvested to evaluate the relative tumor burden. Cryosections of the harvested organs were stained using H&E for histological assessment. RNA from the rest of organs was extracted for qRT-PCR analysis of human hypoxanthine phosphoribosyltransferase (HPRT) mRNA expression.

### Statistical analysis

All statistical analyses were performed using the GraphPad Prism 8.0 software, and all of the data are presented as mean ± standard deviation (SD). Two-tailed Student's t test or one-way ANOVA was used to determine the statistical significance. Survival curves were generated with the Kaplan-Meier method and the log-rank test was used for comparison. A p value of less than 0.05 was considered to be statistically significant.

## Results

### An increase in histone lactylation in ccRCC with inactive VHL

**Figure 1:**
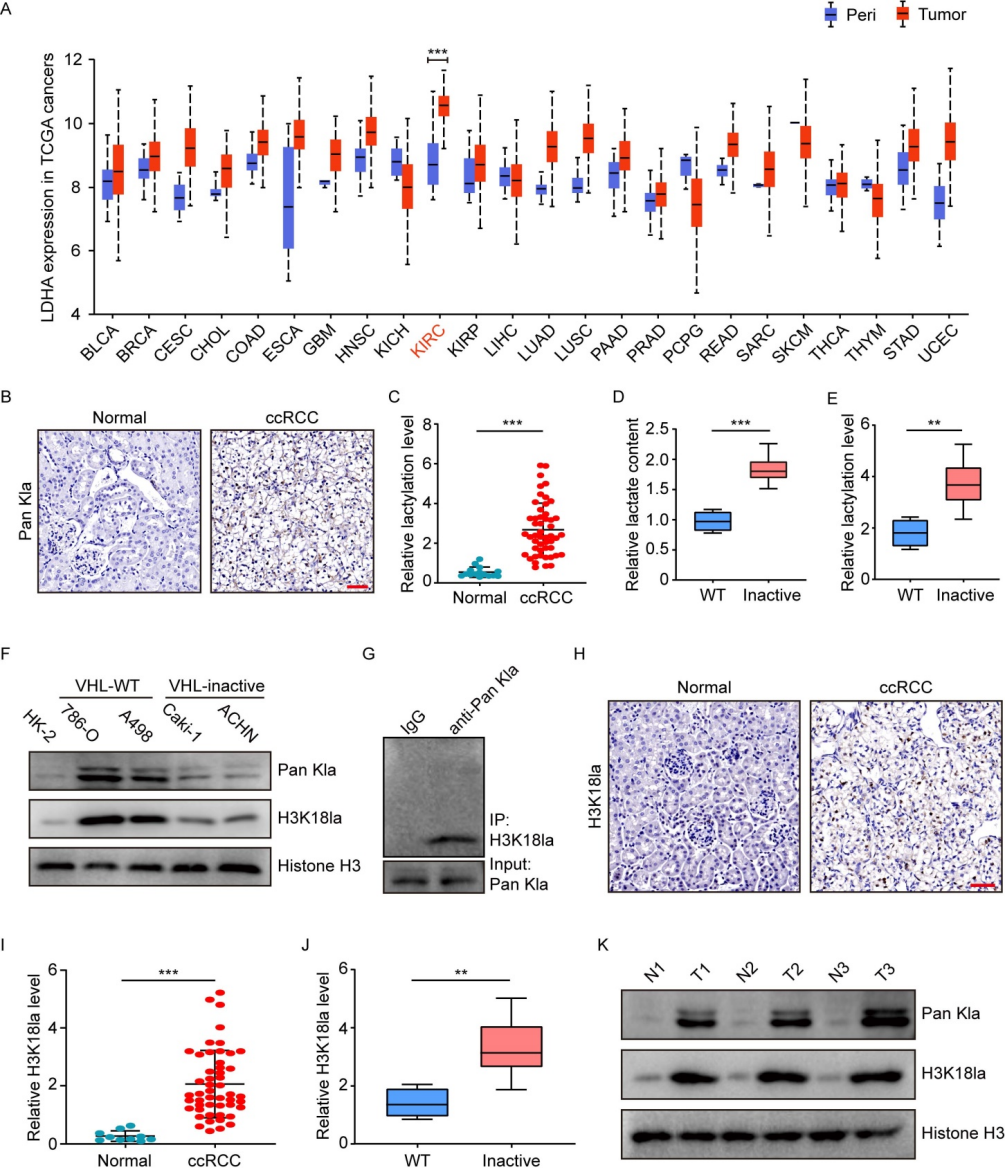
** Elevated histone lactylation levels in VHL-inactive ccRCC tissues and cells.** (**A**) LDHA expression levels in tumors (red box) and peri-tumor tissues (blue box) in 24 human cancer types from TCGA database. (**B**) Representative Immunohistochemistry (IHC) images showing the global histone lactylation (Pan Kla) levels in ccRCC and normal tissues. Scale bar, 50 µm. (**C**) Quantification of global lactylation levels in ccRCC and normal tissues. (**D**) Relative lactate content in ccRCC tumor tissues with wild-type (WT) or inactive VHL. (**E**) Quantification of global lactylation levels in ccRCC tumor tissues with wild-type (WT) or inactive VHL. (**F**) Histone global lactylation and H3K18la levels were measured by Western blot in immortalized renal epithelial cell, VHL inactive (VHL (-)) RCC cell lines and RCC cell lines with endogenous VHL (VHL (+)). (**G**) Immunoprecipitated lactylated proteins were detected by western blot. (**H**) Representative Immunohistochemistry (IHC) images showing the H3K18la levels in ccRCC and normal tissues. Scale bar, 50 µm. (**I**) Quantification of H3K18la levels in ccRCC and normal tissues. (**J**) Quantification of H3K18la levels in ccRCC tumor tissues with wild-type (WT) or inactive VHL. (**K**) Global Lactylation and H3K18la levels were detected in tumor tissues and paired normal tissues by Western blot. Data are presented as mean±SD. **p<0.01, ***p<0.001, by 2-tailed Student's t test (A, C, D, E, I, J). Peri, peri-tumor tissues; KIRC, Kidney Renal Clear Cell Carcinoma in TCGA.

To explore the potential role of histone lactylation in cancers, the Cancer Genome Atlas (TCGA) database was initially used to examine the expression of lactate dehydrogenase (LDHA and LDHB), two key enzymes involved in histone lactylation modification. In comparison with peri-tumor tissues, most of the 24 cancer types in TCGA had overexpressed mRNA levels of LDHA, but not LDHB (Figure 1A and S1A). Among the cancers with increased LDHA, kidney renal clear cell carcinoma (KIRC) is shown to be the top one (Figure 1A). In addition, the National Cancer Institute's Clinical Proteomic Tumor Analysis Consortium (CPTAC) dataset presented a similar pattern of LDHA protein expression to its mRNA trend in TCGA (Figure S1B). Given the link among LDHA, lactate and histone lactylation, these findings suggest the presence of histone lactylation in KIRC.

Subsequently, we tested the histone lactylation levels by immunohistochemical staining of 50 ccRCC and 15 normal kidney tissues. Results demonstrated ccRCC versus normal kidney tissues exhibited significantly higher levels of global histone lactylation (Figure 1B-1C). Stratification of the cohort into patients with and without inactive VHL indicated VHL-inactive versus VHL wild-type ccRCC had significantly more lactate content (Figure 1D), as well as higher levels of global histone lactylation (Figure 1E). These trends were confirmed by western blots in VHL-inactive (786-O and A498) versus VHL wild-type RCC cell lines (Caki-1 and ACHN) (Figure 1F). Immunoprecipitation of lactylated proteins showed the histone H3K18la represented the main target for global histone lactylation (Figure 1G), and H3K18la displayed a similar expression pattern to the global lactylation (Figure 1H-1J). This phenomenon was further reflected in ccRCC tissues compared with the matched adjacent normal tissues (Figure 1K). Together, these results point out to a positive link of histone lactylation with VHL inactivation in ccRCC.

### The causal link between inactive VHL and histone lactylation

**Figure 2:**
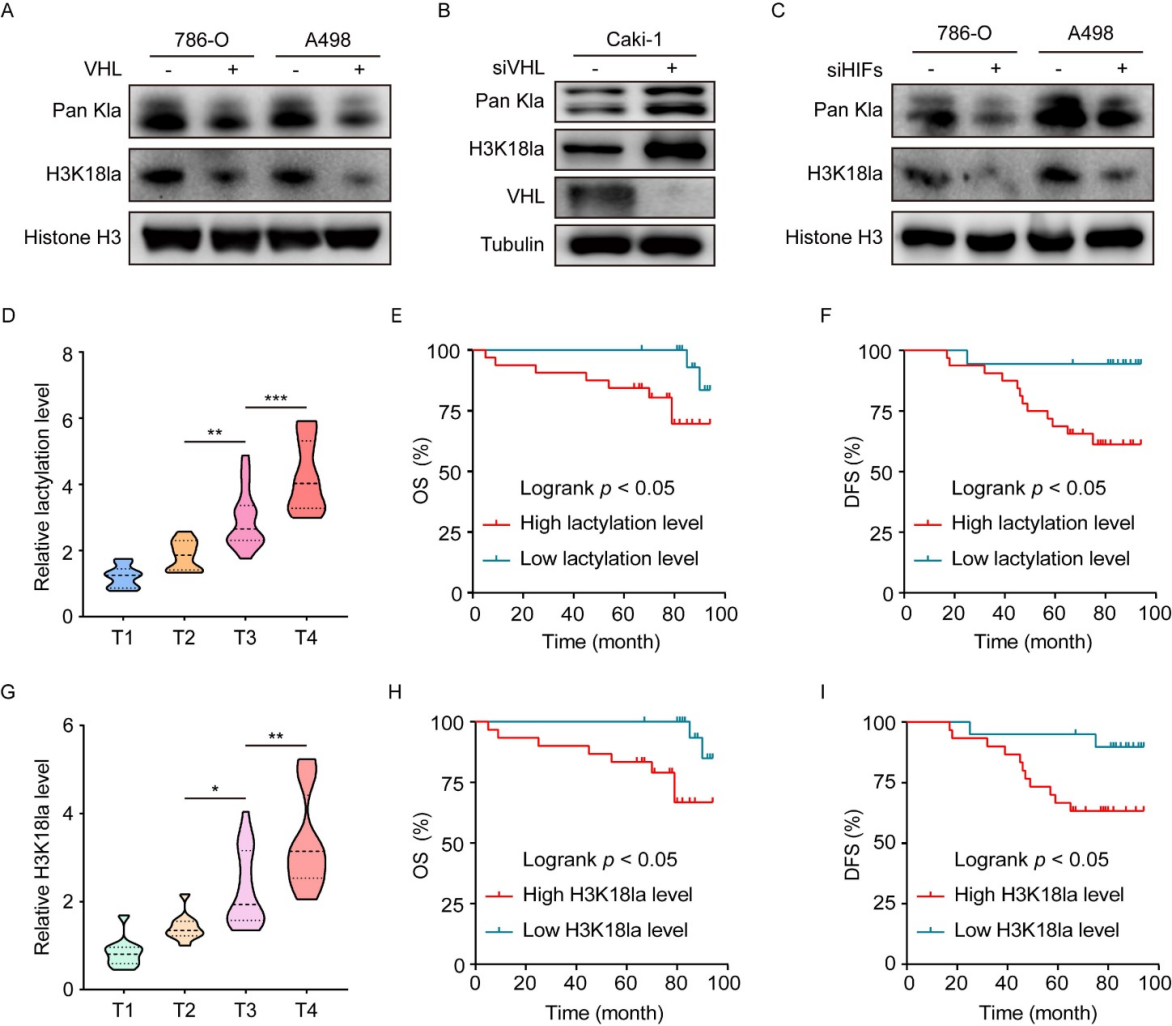
** Histone lactylation is caused by VHL inactivation and its association with clinical outcomes.** (**A**) Western blot showing histone global lactylation and H3K18la levels in WT (-) and VHL-reconstituted (+) 786-O and A498 cells. (**B**) Western blot showing histone global lactylation and H3K18la levels in Caki-1 cells with or without VHL knockdown. (**C**) Western blot showing histone global lactylation and H3K18la levels in 786-O and A498 cells with or without HIF-1α and HIF-2α knockdown. (**D**) Global lactylation levels at different AJCC stages. There were 10 ccRCC samples in T1 stage, 10 in T2 stage, 18 in T3 stage, and 12 in T4 stage. (**E**, **F**) Kaplan-Meier analysis of overall survival (**E**) and disease-free survival (**F**) in ccRCC patients with low (n = 18) and high (n = 32) global lactylation levels. (**G**) H3K18la levels at different AJCC stages. There were 10 ccRCC samples in T1 stage, 10 in T2 stage, 18 in T3 stage, and 12 in T4 stage. (**H, I**) Kaplan-Meier analysis of overall survival (**H**) and disease-free survival (**I**) in ccRCC patients with low (n=20) and high (n=30) H3K18la levels. Data are presented as mean±SD. *p<0.05, **p<0.01, ***p<0.001, by 1-way ANOVA (D, G) or Log-rank test (E, F, H, I).

To verify whether the presence of histone lactylation is determined by inactive VHL, we reconstituted VHL expression in VHL-deficient 786-O and A498 cells or knocked down VHL in VHL wild-type Caki-1 cells using siRNA. Western blots indicated that reconstitution of VHL in 786-O and A498 cells significantly decreased the levels of global lactylation, as well as H3K18la (Figure 2A and S2A), while VHL knockdown in Caki-1 cells provided an opposite tendency (Figure 2B). These findings indicated loss of VHL triggered histone lactylation. Given the well-established link of VHL with HIFs, we set out to test the contribution of HIFs to inactive VHL-triggered histone lactylation. To this end, HIFs were knocked down using a siRNA pool in VHL-deficient 786-O and A498 cells (Figure S2B). Results demonstrated that loss of HIFs effectively abrogated the VHL loss-induced increase in global lactylation and H3K18la levels (Figure 2C). These data indicate VHL inactivation induced histone lactylation in a HIFs-dependent manner.

We next assessed the clinical importance of histone lactylation in a cohort of ccRCC patients. Results showed that the global lactylation level increased with more advanced AJCC stages (Figure 2D), and Kaplan-Meier analysis revealed patients with high versus low levels of global lactylation levels had poorer overall survival (OS) (Figure 2E). In addition, H3K18la levels positively correlated with AJCC stages (Figure 2G), and patients with high versus low levels of H3K18la exhibited worse OS (Figure 2H). Clinical significance of disease-free survival (DFS) was shown to have similar trends (Figure 2F and 2I). These findings suggest that inactive VHL-triggered histone lactylation might play functions in ccRCC progression, thereby contributing to a poor patient prognosis.

### The effects of histone lactylation on the progression of ccRCC

**Figure 3:**
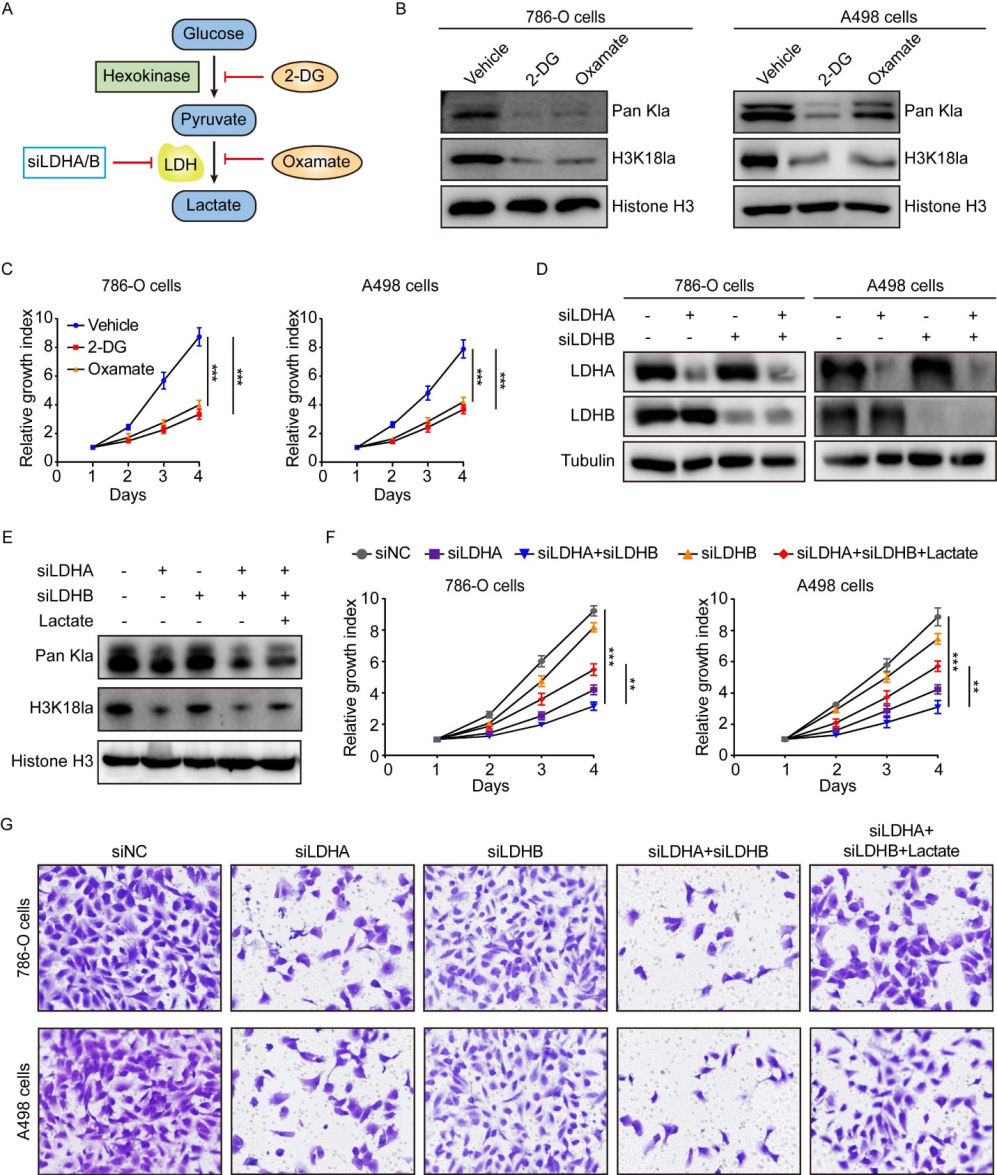
** Inhibition of histone lactylation impairs proliferation and migration ability of ccRCC cells.** (**A**) Schematic diagram of methods to inhibit histone lactylation. (**B**) Western blot showing global lactylation and H3K18la levels in 786-O (left panel) and A498 (right panel) cells treated with 4mM 2-DG or 8mM oxamate for 24h. (**C**) CCK-8 assay showing the proliferation ability of 786-O (left panel) and A498 (right panel) cells treated with 4mM 2-DG or 8mM oxamate. (**D**) Western blot showing the expression levels of LDHA and LDHB in 786-O (left panel) and A498 (right panel) cells with LDHA and LDHB knockdown. (**E**) Representative Western blot showing global lactylation and H3K18la levels in ccRCC cells with LDHA/LDHB knockdown and supplemented with 5mM lactate. (**F**) CCK-8 assay showing the proliferation ability of 786-O (left panel) and A498 (right panel) cells with LDHA/LDHB knockdown and supplemented with 5mM lactate. (**G**) Transwell assay showing the migration ability of 786-O and A498 cells with LDHA/LDHB knockdown and supplemented with 5mM lactate. Data are presented as mean±SD. **p<0.01, ***p<0.001, by 1-way ANOVA (C, F).

In light of our above findings, we set out to explore the biological functions of histone lactylation in tumor progression. As described previously 15, 18, histone lactylation was blocked by glycolytic inhibitors (2-deoxy-D-glucose (2-DG) and oxamate) or siRNAs for LDHA and LDHB (Figure 3A). As anticipated, glycolytic inhibitors reduced intracellular lactate levels in a concentration-dependent manner (Figure S3A-S3B). In line with the decreased lactate levels, the levels of global lactylation and H3K18la were efficiently inhibited by glycolytic inhibitors (Figure 3B). More importantly, a decrease in histone lactylation was able to suppress the growth of ccRCC cells (Figure 3C).

Since 2-DG and oxamate are likely to introduce other effects independent of inhibiting lactate production and lactylation, we further inhibited histone lactylation by using siRNA specific for LDHA and LDHB in ccRCC cells (Figure 3D). We found depletion of LDHA, but not LDHB, significantly inhibited histone lactylation and simultaneous silencing LDHA and LDHB did not significantly boost the effect as compared to silencing LDHA alone (Figure 3E). This corroborated with the TCGA data that LDHA but not LDHB was significantly up-regulated in KIRC. CCK-8 assays demonstrated LDHA depletion or combined depletion of LDHA and LDHB repressed the growth of 786-O and A498 cells (Figure 3F). Transwell assays indicated silencing LDHA or both LDHA and LDHB inhibited the migration of ccRCC cells (Figure 3G). Moreover, the addition of exogenous lactate to LDHA/LDHB-deficient cells was able to restore histone lactylation levels of ccRCC cells (Figure 3E), thereby eliciting recovered proliferation and migration ability (Figure 3F-3G). Together, our results uncover a critical role of histone lactylation in ccRCC progression.

### The transcription activation of platelet-derived growth factor receptor β by histone lactylation

**Figure 4:**
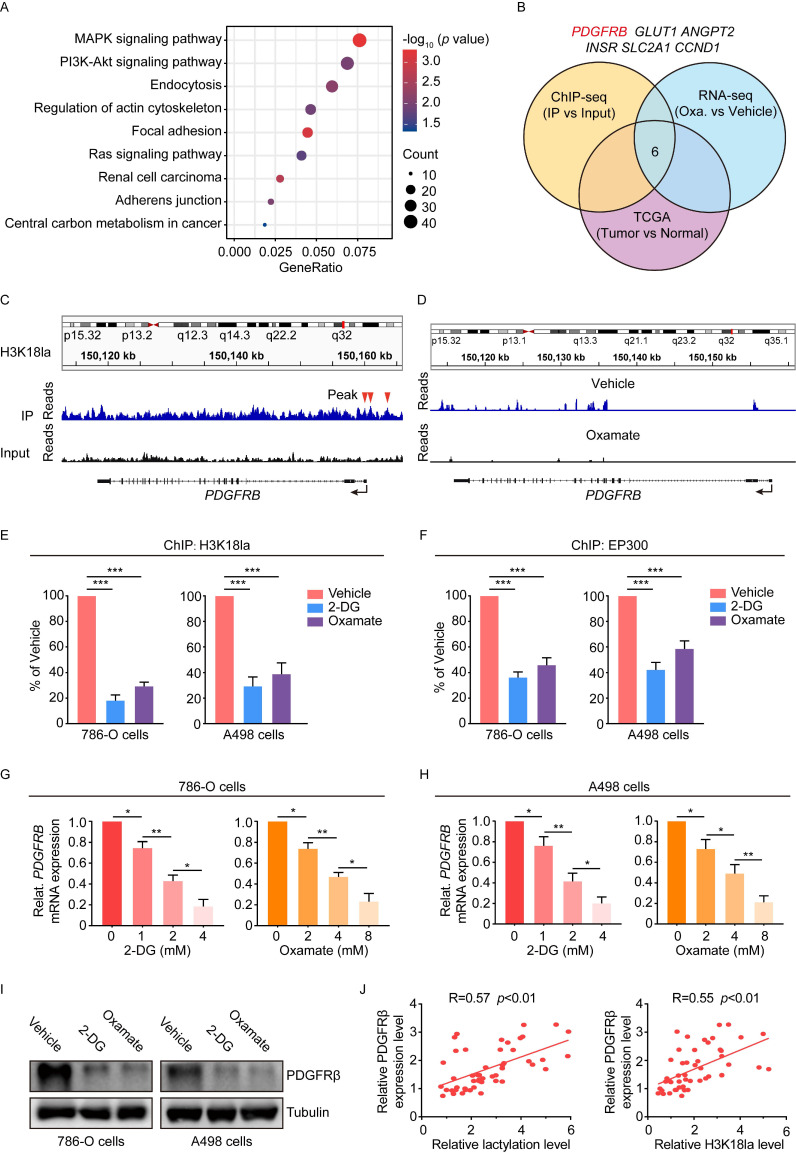
** Histone lactylation transcriptionally activates PDGFRβ.** (**A**) KEGG analysis of H3K18la peaks revealed by chromatin immunoprecipitation sequencing (ChIP-seq) of 786-O cells. (**B**) Strategy to identify potential specific downstream targets of histone H3K18la modification. (**C**) Representative IGV tracks showing enriched H3K18la modification in PDGFRB promotor by ChIP-seq. Arrows are the H3K18la peaks at the PDGFRB promotor. (**D**) Representative IGV tracks showing decreased PDGFRB expression upon oxamate treatment by RNA-seq. (**E**) ChIP-qPCR analysis for H3K18la status at the PDGFRB promotor of 786-O (left panel) an A498 (right panel) cells treated with 4mM 2-DG or 8mM oxamate for 24h. (**F**) ChIP-qPCR analysis for EP300 status at the PDGFRB promotor of 786-O (left panel) an A498 (right panel) cells treated with 4mM 2-DG or 8mM oxamate for 24h. (**G, H**) mRNA expression levels of PDGFRB in 786-O cells (**G**) and A498 cells (**H**) treated with indicated concentrations of 2-DG (left panel) or oxamate (right panel) for 24h as determined by RT-qPCR. (**I**) Western blot showing the expression of PDGFRβ in 786-O (left panel) and A498 (right panel) cells treated with 4mM 2-DG or 8mM oxamate for 24h. (**J**) Correlation between PDGFRβ expression levels and global lactylation (left panel) or H3K18la (right panel) levels in ccRCC patients. Data are presented as mean±SD. *p<0.05, **p<0.01, ***p<0.001, by 1-way ANOVA (E, F, G, H) or Person's correlation analysis (J).

Subsequently, we tried to understand how histone lactylation promotes ccRCC progression. To this end, chromatin immunoprecipitation with sequencing (ChIP-seq) in 786-O cells using anti-H3K18la antibody was performed. ChIP-seq data were able to identify multiple sequencing reads enriched in promoter regions (Figure S4A), and KEGG analysis revealed that H3K18la-regulated genes were involved in oncogenic signaling pathways such as MAPK, PI3K-Akt, and Ras (Figure 4A). By overlapping the gene sets among ChIP-seq, TCGA-KIRC and 786-O cells treated with oxamate, 6 genes were noted (Figure 4B). Among them, the platelet-derived growth factor receptor β (PDGFRB) promoter in the genomic position was identified to have a marked enrichment of H3K18la peaks (Figure 4C). In agreement, oxamate treatment led to a significant decrease in the PDGFRB expression (Figure 4D). In TCGA database, PDGFRB was upregulated in KIRC, but not in KICH and KIRP (Figure S4B). Moreover, ChIP-qPCR assays confirmed that H3K18la was enriched at the PDGFRB promoter, which was reduced by glycolytic inhibitors (Figure 4E). In addition, the binding of histone lactylation writer EP300 to the PDGFRB promoter was dramatically decreased by glycolysis inhibitor (Figure 4F), despite that the global level of EP300 remained unchanged (Figure S4C). H3K27ac level at the PDGFRB promoter, however, was shown to have a slight decline under such treatment (Figure S4D). Consistent with the ChIP results, glycolytic inhibitors suppressed both mRNA and protein expression levels of PDGFRβ (Figure 4G-4I). However, the mRNA stability of PDGFRβ was shown to be unchanged upon glycolytic inhibitors (Figure S4E). These findings suggested the downregulation of PDGFRβ was attributable to the transcription reduction resulting from reduced H3K18la in its promoter. Clinically, PDGFRβ expression was found to positively correlate with both global lactylation and H3K18la levels in ccRCC specimens (Figure 4J). Collectively, these data indicated histone lactylation transcriptionally activates the expression of PDGFRβ.

### The contribution of PDGFRβ to histone lactylation-mediated tumor progression

**Figure 5:**
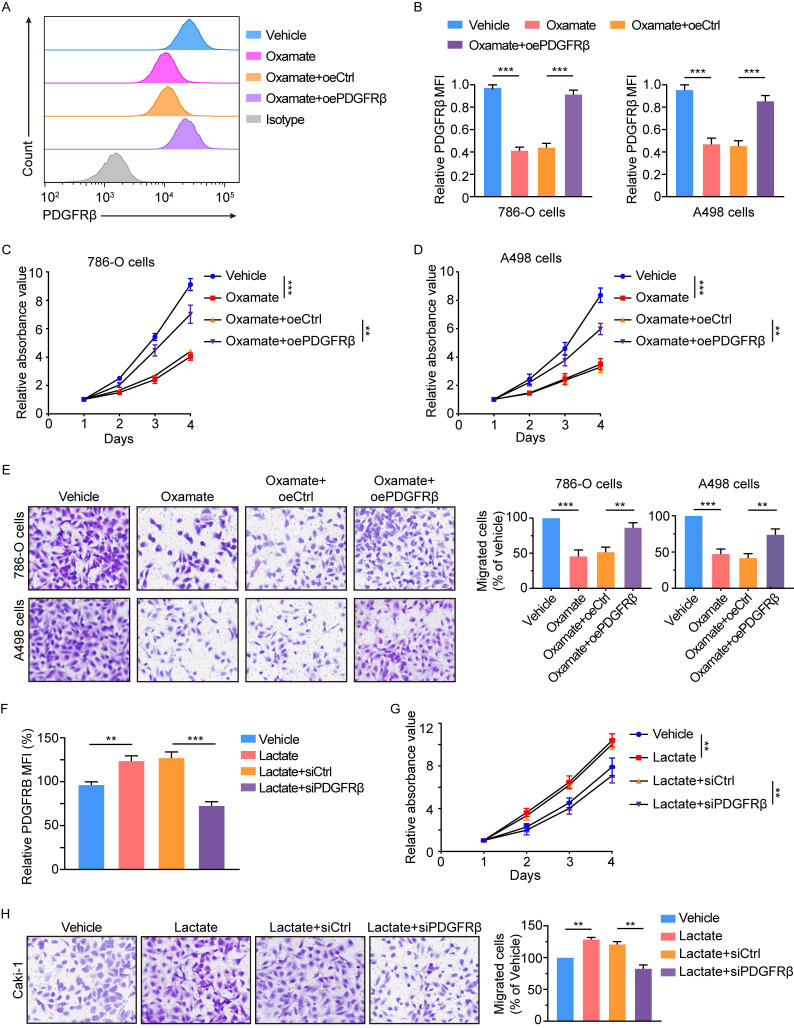
** Histone lactylation enhances tumor proliferation and migration largely dependent on PDGFRβ pathway.** (**A**) Representative flow cytometry histogram of PDGFRβ in ccRCC cells with or without PDGFRβ overexpression. Cells were treated with 8mM oxamate for 24 h. Overexpressing PDGFRβ restored the oxamate-induced down-regulation of PDGFRβ. (**B**) Quantification of relative PDGFRβ MFI in 786-O (left panel) and A498 (right panel) cells with or without PDGFRβ overexpression treated with oxamate. (**C**, **D**) CCK-8 assay showing the proliferation ability of 786-O (**C**) and A498 (**D**) cells with or without PDGFRβ overexpression treated with oxamate. (**E**) Transwell assay showing the migration ability of 786-O and A498 cells with or without PDGFRβ overexpression treated with oxamate. (**F**) Quantification of relative PDGFRβ MFI in Caki-1 cells with or without PDGFRβ knockdown. Cells were treated with 5mM lactate for 24 h. PDGFRβ knockdown inhibited the lactate-induced up-regulation of PDGFRβ. (**G**) CCK-8 assay showing the proliferation ability of Caki-1 cells with or without PDGFRβ knockdown treated with lactate. (**H**) Transwell assay showing the migration ability of Caki-1 cells with or without PDGFRβ knockdown treated with lactate. Data are presented as mean ± SD. **p<0.01, ***p<0.001, by 1-way ANOVA (B, C, D, E, F, G, H). oe, overexpression.

To determine whether histone lactylation exerts its oncogenic role through PDGFRβ signaling, we overexpressed PDGFRβ in oxamate-treated 786-O and A498 cells (Figure 5A-5B). Results showed ectopic expression of PDGFRβ significantly abolished oxamate-induced inhibition on the proliferation and migration of 786-O and A498 cells (Figure 5C-5E). Moreover, we also tested the effects of PDGFRβ silencing in Caki-1 cells that had been pretreated with lactate. We found disruption of PDGFRβ signaling significantly abolished the lactate-elicited increase in the proliferation and migration of Caki-1 cells (Figure 5F-5H). Together, we conclude histone lactylation facilitates ccRCC progression through activating PDGFRβ signaling.

### A feedback loop between PDGFRβ signaling and histone lactylation

**Figure 6:**
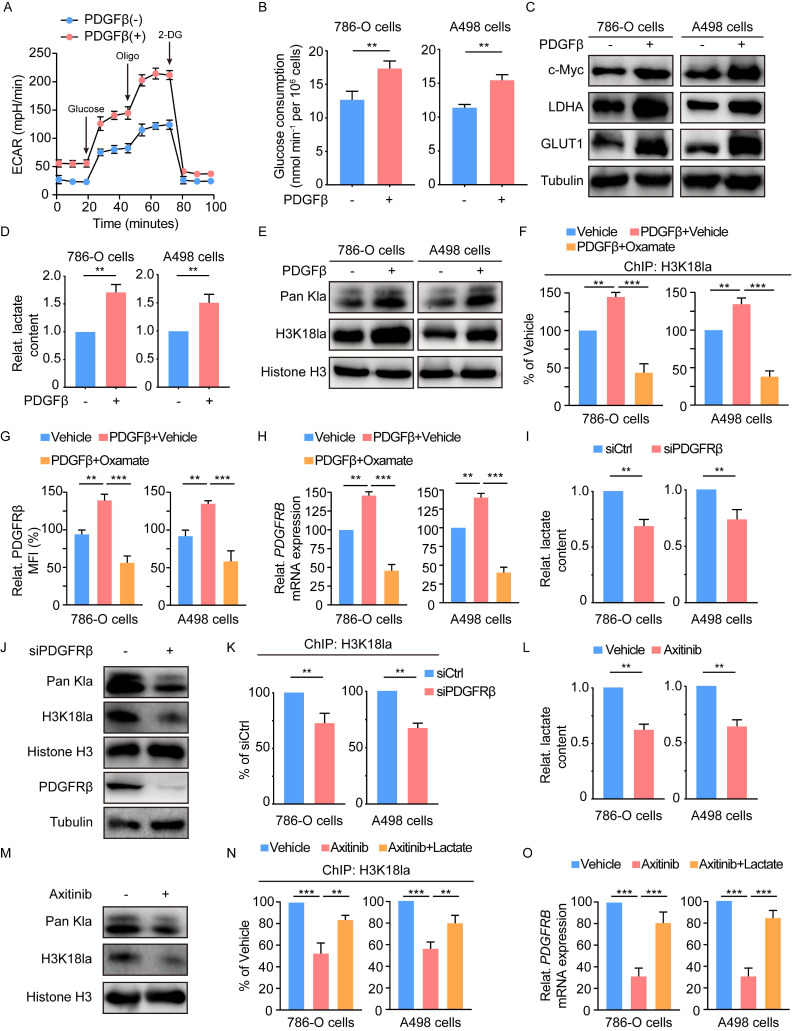
** Histone lactylation-PDGFRβ signaling forms a feedback loop in ccRCC.** (**A**) Extracellular acidification rate (ECAR) of 786-O cells in the presence or absence of PDGFβ stimulation. (**B**) Glucose consumption of 786-O (left panel) and A498 (right panel) cells incubated with no serum DMEM in the presence or absence of PDGFβ stimulation. (**C**) Western blot showing the expression of glycolysis-related genes in 786-O (left panel) and A498 (right panel) cells incubated with no serum DMEM in the presence or absence of PDGFβ stimulation. (**D**) Relative intracellular lactate levels of 786-O (left panel) and A498 (right panel) cells incubated with no serum DMEM in the presence or absence of PDGFβ stimulation. (**E**) Western blot showing the global lactylation and H3K18la levels of 786-O (left panel) and A498 (right panel) cells incubated with no serum DMEM in the presence or absence of PDGFβ stimulation. (**F**) ChIP-qPCR analysis for H3K18la status at the PDGFRB promoter of 786-O (left panel) an A498 (right panel) cells treated with PDGFβ stimulation and 8mM oxamate. (**G**) Quantification of relative PDGFRβ MFI in 786-O (left panel) and A498 (right panel) cells treated with PDGFβ stimulation and oxamate. (**H**) mRNA expression levels of PDGFRB in 786-O cells (left panel) and A498 (right panel) cells treated with PDGFβ stimulation and oxamate. (**I**) Relative intracellular lactate levels of 786-O (left panel) and A498 (right panel) cells with PDGFRβ knock down. (**J**) Representative Western blot showing the global lactylation and H3K18la levels of ccRCC cells with PDGFRβ knock down. (**K**) ChIP-qPCR analysis for H3K18la status at the PDGFRB promoter of 786-O (left panel) an A498 (right panel) cells with PDGFRβ knock down. (**L**) Relative intracellular lactate levels of 786-O (left panel) and A498 (right panel) cells treated with 2μM Axitinib. (**M**) Representative Western blot showing the global lactylation and H3K18la levels of ccRCC cells treated with Axitinib. (**N**) ChIP-qPCR analysis for H3K18la status at the PDGFRB promoter of 786-O (left panel) an A498 (right panel) cells treated with Axitinib and 5mM lactate. (**O**) mRNA expression levels of PDGFRB in 786-O cells (left panel) and A498 (right panel) cells treated with Axitinib and lactate. Data are presented as mean±SD. **p<0.01, ***p<0.001, by 2-tailed Student's t test (B, D, I, K, L) or 1-way ANOVA (F, G, H, N, O).

Previous works have indicated a link of RTK signaling with glycolysis 19, 20. Given the well-established association of glycolysis with histone lactylation 15, we inquired whether PDGFRβ signaling could stimulate histone lactylation. To this end, 786-O and A498 cells were treated with recombinant PDGFβ. In response to PDGFβ stimulation, significant increases were found in the extracellular acidification rate (ECAR) (Figure 6A), glucose consumption (Figure 6B), the expression of glycolysis-related genes (c-MYC, LDHA and GLUT1) (Figure 6C), and the lactate production (Figure 6D). More importantly, the addition of PDGFβ resulted in an obvious increase in histone lactylation (Figure 6E). ChIP-qPCR assays revealed the enrichment of H3K18la at the PDGFRB promoter upon PDGFβ stimulation was in a lactate-dependent manner (Figure 6F). Paralleled with these findings, the increase in PDGFRβ mRNA and protein levels by PDGFβ stimulation could be abolished by oxamate (Figure 6G-6H). In addition, knock down of PDGFRβ led to a significant decrease in lactate production (Figure 6I), histone lactylation level (Figure 6J), as well as the enrichment of H3K18la at the PDGFRB promoter (Figure 6K). Treatment with a clinically applicable PDGFRβ inhibitor Axitinib dramatically reduced intracellular lactate (Figure 6L) and histone lactylation levels (Figure 6M). In line with PDGFRβ knockdown, H3K18la level on PDGFRB promoter and the transcription of PDGFRβ mRNA were markedly decreased, which could be partially reversed by addition of lactate (Figure 6N-6O). These results indicate PDGFRβ signaling stimulates histone lactylation, and point out to a positive feedback loop between histone lactylation and PDGFRβ signaling.

### Therapeutic efficacy of blocking histone lactylation-PDGFRβ loop

**Figure 7:**
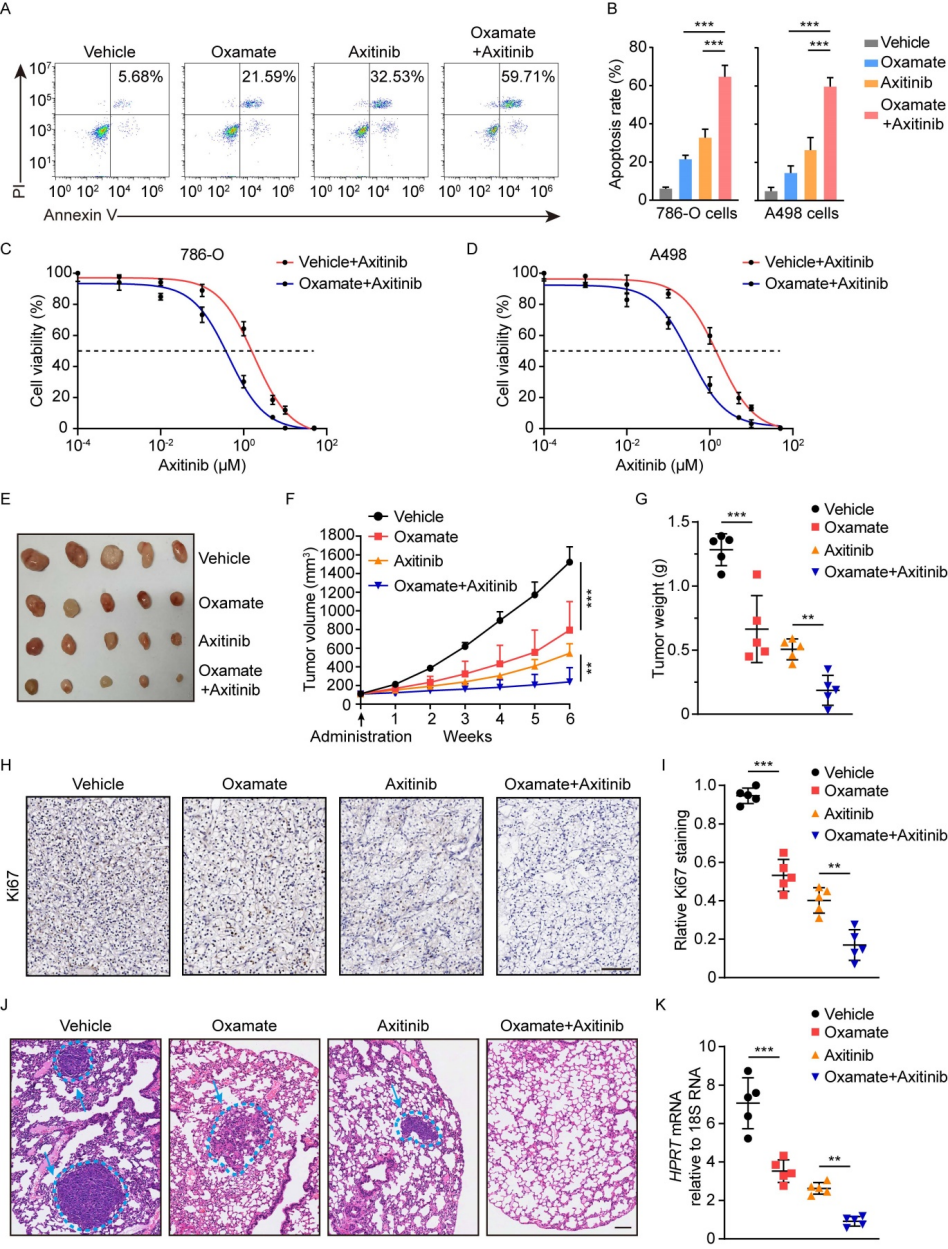
** Therapeutic potential of inhibiting histone lactylation-PDGFRβ feedback loop.** (**A**,** B**) Representative Annexin V-PI (propidium iodide) staining **(A)** and quantification (**B**) of ccRCC cells treated with 8mM oxamate and 2μM Axitinib. (**C**,** D**) IC50 values of 786-O (**C**) and A498 (**D**) cells treated with 8mM oxamate and 2μM Axitinib. (**E-G**) The combination of oxamate and Axitinib remarkedly inhibited the growth of tumors in ccRCC PDX mice. Tumor images (**E**), tumor growth curves (**F**) and tumor weights (**G**) were shown. (**H**,** I**) Representative Immunohistochemistry (IHC) staining (**H**) and quantification (**I**) of Ki67 in ccRCC PDX mice with indicated treatment. Scale bar, 100 µm. (**J**,** K**) The combination of oxamate and Axitinib significantly inhibited ccRCC in vivo lung micro-metastasis established with 786-O cells. HE staining (**J**) and expression levels of human HPRT mRNA relative to mouse 18S rRNA (**K**) in the lungs of pulmonary metastasis models were shown. Scale bar, 100µm. Data are presented as mean±SD. **p<0.01, ***p<0.001, by 1-way ANOVA (B, F, G, I, K).

We then determined the therapeutic potential of blocking histone lactylation and PDGFRβ. In vitro experiments demonstrated that either target correction of aberrant histone lactylation by oxamate or inhibition of PDGFRβ by Axitinib could induce massive apoptosis, but these effects were reinforced by the combined inhibition of histone lactylation and PDGFRβ (Figure 7A-7B). Treatment with oxamate in 786-O and A498 cells significantly increased their sensitivities to Axitinib, as indicated by decreased IC50 (Figure 7C-7D). Using a ccRCC PDX mouse model, either Axitinib or oxamate dramatically decreased tumor growth, as shown by tumor size and weight (Figure 7E-7G). IHC staining indicated that expression of Ki67 was markedly reduced upon Axitinib or oxamate treatment (Figure 7H-7I). The combined treatment with Axitinib or oxamate significantly reinforces the therapeutic efficacy (Figure 7E-7I). In addition, the therapeutic efficacy of spontaneously blocking histone lactylation and PDGFRβ was further confirmed by tail-vein injection lung metastasis mouse models (Figure 7J-7K). Collectively, our findings underscore the significance of blocking the histone lactylation-PDGFRβ loop in ccRCC treatment.

## Discussion

**Figure 8:**
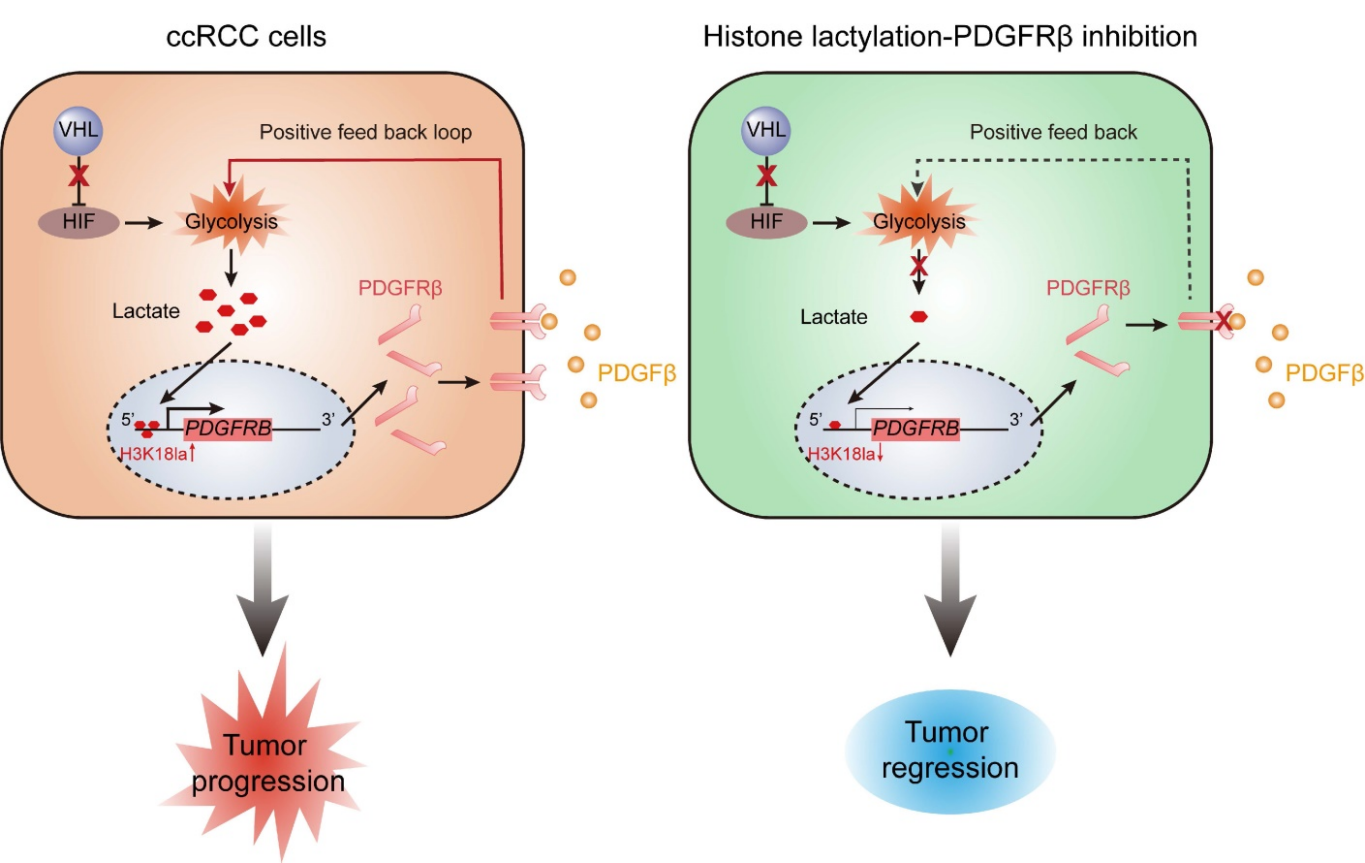
** Schematics diagram depicting the key findings of this study.** A VHL inactivation triggered histone lactylation-PDGFRβ signaling positive feedback loop promotes ccRCC progression, and target correction of this vicious circle could combat ccRCC.

Mutations in genes encoding epigenetic modification enzymes (such as PBRM1, SETD2, BAP1, KDM5C, KDM6A, and MLL2) have been shown to play vital roles in the initiation and development of RCC 21-23. Of interest, recent research has indicated a novel form of histone modification known as lactylation, which was orchestrated by glycolysis-derived lactate and had the ability to directly stimulate gene transcription 15. Given the well-established link of VHL/HIFs signaling with lactate in ccRCC 24, we hypothesised an abnormity in histone lactylation might contribute to the pathogenesis of ccRCC. Support for this possibility comes from this report in which it is shown that VHL-inactive ccRCC has a significant increase in histone lactylation, which promotes the progression of ccRCC (Figure 8). The present work, to our knowledge, is the first study that comprehensively elucidates the role and targeted therapeutic potential of histone lactylation in ccRCC.

It is well established that metabolic dysfunction represents a key hallmark of cancer 25. Cancer cells reprogram metabolic process to support rapidly proliferation and survival. Known as an energy source and metabolic by-product, lactate and has been identified to correlate with multiple biological functions of cancers 26, and is mainly produced by two enzymes LDHA and LDHB 27. They exert different roles in lactate production. LDHA has an affinity for pyruvate and preferentially convert pyruvate to lactate, whereas LDHB exhibits an affinity for lactate, preferentially converting lactate to pyruvate. TCGA database demonstrates a different expression pattern of LDHA and LDHB, suggesting a distinct link of LDHA and LDHB with histone lactylation. This study further supports this notion by demonstrating that silencing LDHA resulted in a dramatical decrease in histone lactylation in 786-O and A498 cells, whereas only a slight change was found upon silencing LDHB. Whether the different contributions of LDHA and LDHB to histone lactylation is a general feature in cancers requires further efforts.

The discovery of histone lactylation links lactate to gene expression in physiology and disease 28. Yu and colleagues indicated lactate-triggered histone lactylation stimulated YTHDF2 expression, which subsequently recognized the m6A-modified mRNAs of PER1 and TP53 and promoted their degradation, thereby facilitating ocular melanoma oncogenesis 18. However, TCGA-KIRC database found comparable levels of YTHDF2 in tumor versus normal tissues in ccRCC. In line with this, our ChIP-seq results did not identify a significant association between histone lactylation and YTHDF2. These insights imply one gene might be regulated by histone lactylation in a specific system, and histone lactylation in one system may affect different genes. We here uncover that histone lactylation activates the transcription of PDGFRβ in ccRCC. In VHL-inactive ccRCC, histone lactylation is likely to act as a bridge between VHL deficiency and PDGFRβ hyperactivation to support tumor progression. Together, this work presents data for a better understanding of the interactions among metabolic reprogramming, epigenetic modification and cancer pathologies.

Activation of receptor tyrosine kinases has been reported to correlate with enhanced glycolysis 19. As one type of transmembrane receptor tyrosine kinases, PDGFRβ was identified as a key target gene of histone lactylation in ccRCC 29. Upon binding with its corresponding ligand, PDGFRβ is phosphorylated, internalized and activates multiple docking sites for the downstream signal transduction molecules 30. An increase in the expression of PDGFRβ was seen in multiple tumors, including gastrointestinal tumor, lung cancer, breast cancer, hepatocellular carcinoma, and pancreatic cancer 31. Moreover, PDGFRβ signaling was identified to facilitate cell proliferation, metastasis, and angiogenesis of tumors by activating PI3K/AKT and Ras/MAPK pathways 32. Our results found PDGFβ stimulation in ccRCC cells led to an increase in glycolysis, lactate production, as well as histone lactylation, which in turn activated PDGFRβ transcription, thereby forming a vicious cycle that further accelerates the progression of ccRCC. This work presents an alternative mechanism for the significant involvement of PDGFRβ in tumor progression.

In the current clinical practice, PDGFRβ inhibitor Axitinib have been successfully introduced into the treatment of metastatic RCC 33, 34. However, durable clinical responses and long-term remission are observed in only a small portion of patients, and the long-term prognosis remains poor for relapsed and metastatic patients 35, 36. Thus, there is an urgent clinical need for novel treatment options. Given the contribution of the positive feedback loop between PDGFRβ and histone lactylation to the pathogenesis of ccRCC, we designed a combined therapeutic approach for this cancer. Results demonstrated combined inhibition of PDGFRβ signaling and histone lactylation had a synergistical efficacy in combating ccRCC. This strategy has reinforced the rational of Axitinib in clinical use, and is of great potential to seek better therapeutic options and improve clinical outcomes of patients with ccRCC.

In summary, this work indicated VHL inactivation participates in the pathogenesis of ccRCC through triggering histone lactylation, which initiates a positive feedback loop between histone lactylation and PDGFRβ signaling (Figure 8). Our results define a new manner on how inactive VHL affects tumor progression and provide new perspectives for the therapeutic strategies for patients with ccRCC.

## Supplementary Material

Supplementary figures and methods.

## Abbreviations

VHL: von Hippel-Lindau
ccRCC: clear cell renal cell carcinoma
PDGFRβ: platelet-derived growth factor receptor β
PDX: patient derived xenograft
SD: standard deviation
