# Supplementary material for: A Positive Feedback Loop between Inactive VHL-Triggered Histone Lactylation and PDGFRβ Signaling Drives Clear Cell Renal Cell Carcinoma Progression
Source: Int J Biol Sci. 2022 May 13;18(8):3470–83. doi: 10.7150/ijbs.73398 (PMC9134910; doi:10.7150/ijbs.73398)

Supplementary materials for

A Positive Feedback Loop between Inactive VHL-Triggered Histone Lactylation and PDGFRβ Signaling Drives Clear Cell Renal Cell Carcinoma Progression

Author: Jiefeng Yang, Li Luo, Chongyu Zhao, Xiyuan Li, Zimo Wang, Ziwei Zeng, Xin Yang, Xiaobin Zheng, Haiqing Jie, Liang Kang, Shujuan Li, Shuang Liu, Chi Zhou, Huashan Liu

Correspondence:

Huashan Liu, MD, PhD

Email: liuhshan@mail2.sysu.edu.cn

Chi Zhou, MD, PhD

Email: zhouchi2@mail2.sysu.edu.cn

Shuang Liu, MD, PhD

Email: liushuang@sysucc.org.cn

This PDF file includes:

Figure S1-4

Supplementary Methods

Uncropped versions of western blot images

Full view of lung micro-metastasis

Figure S1

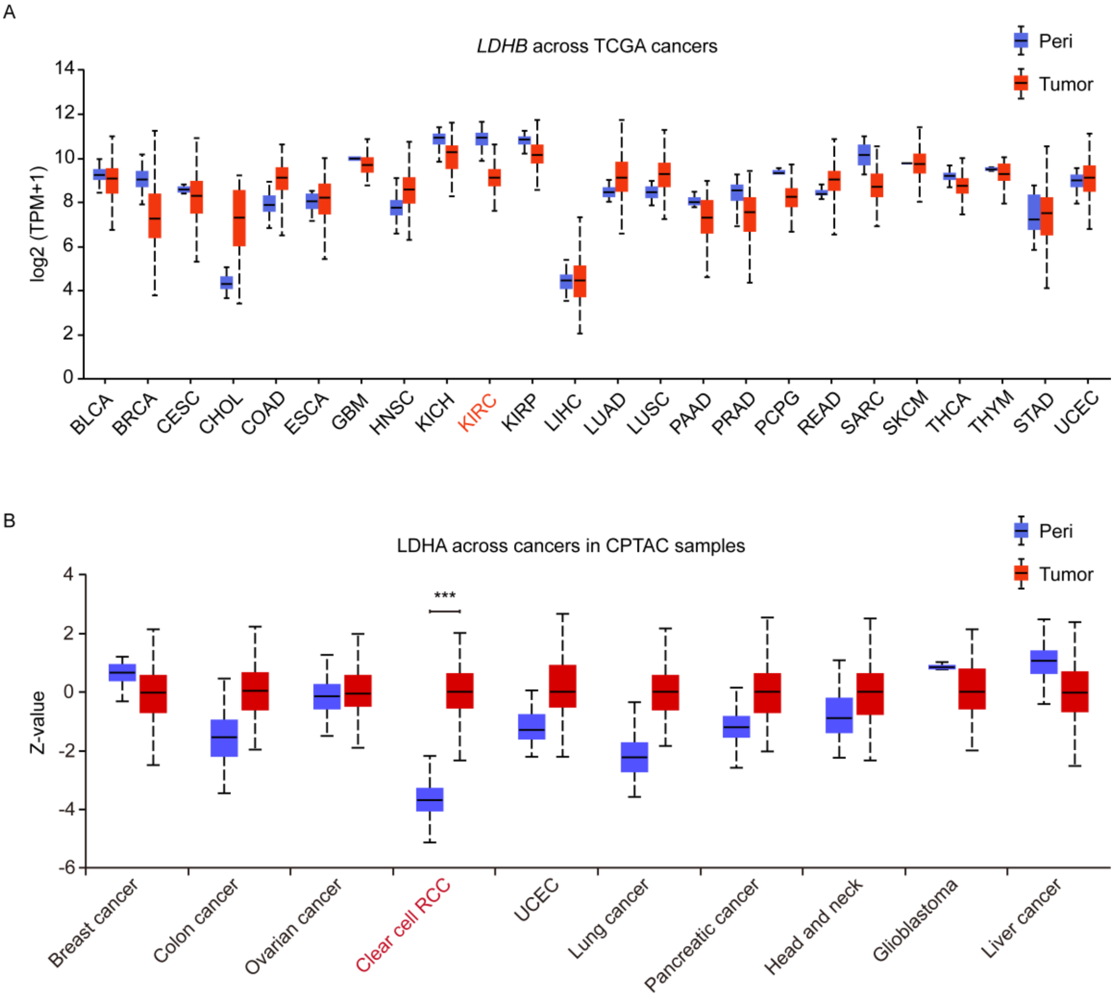

Figure S1. Expression levels of LDHB and LDHA in human cancers. (**A**) *LDHB* expression levels in tumors (red box) and peri-tumor tissues (blue box) in 24 human cancer types from TCGA database. (**B**) Mass-spectrometry-based proteomic expression levels of LDHA in tumor and peri-tumor tissues in CPTAC dataset. Data are presented as mean±SD. ***p<0.001, by 2-tailed Student's t test (B).

Figure S2

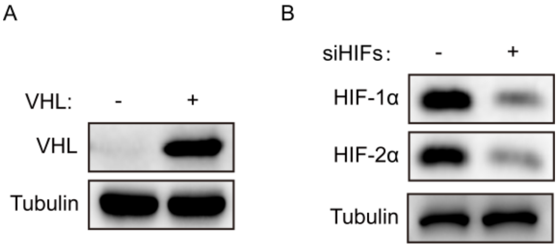

Figure S2. VHL inactive contributed to histone lactylation. (**A**) Representative Western blot showing the reconstitution of VHL in VHL inactive cells. (**B**) Representative Western blot showing the knockdown of HIF-1α and HIF-2α in VHL inactive cells.

Figure S3

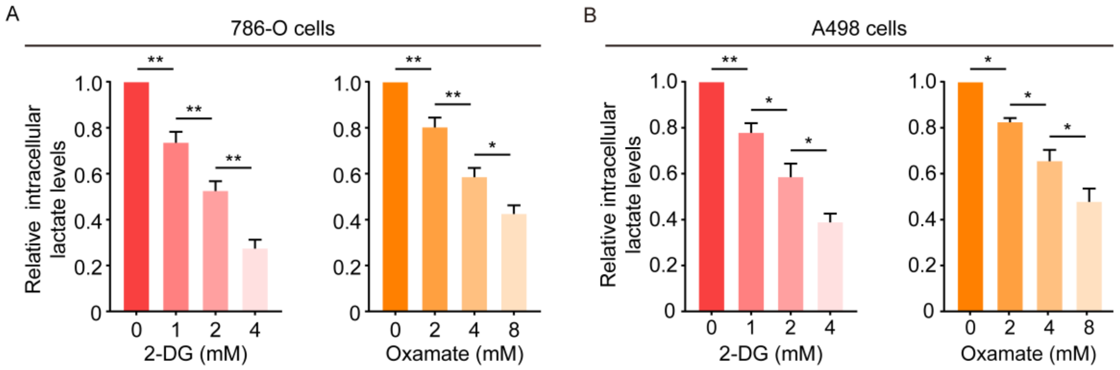

Figure S3. Inhibition of histone lactylation impairs proliferation and migration ability of ccRCC cells. (**A**, **B**) Relative intracellular lactate levels of 786-O cells (**A**) and A498 cells (**B**) treated with indicated concentrations of 2-DG (left panel) or oxamate (left panel) for 24 h. Data are presented as mean±SD. *p<0.05, **p<0.01, by 1-way ANOVA (A, B).

Figure S4

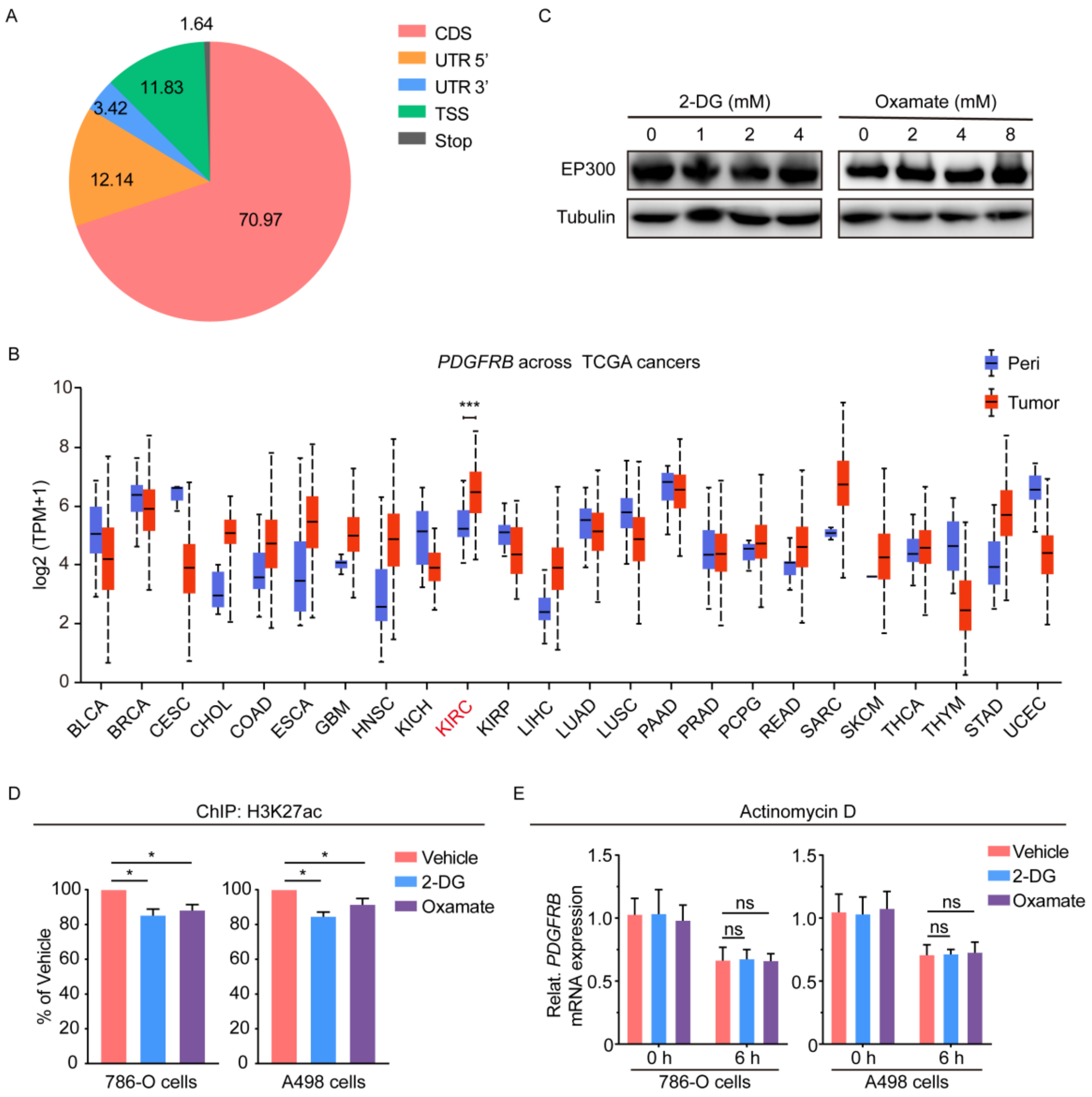

Figure S4. Histone lactylation up-regulates expression of PDGFRβ. (**A**) Distribution of peaks enriched by H3K18la on the genome of 786-O cells. (**B**) *PDGFRB* expression levels in tumors and peri-tumor tissues from TCGA database. (**C**) Western blot showing the expression of EP300 in ccRCC cells with the indicated treatments. (**D**) ChIP-qPCR analysis for H3K27ac status at the *PDGFRB* promotor in 786-O and A498 cells with the indicated treatments. (**E**) *PDGFRB* mRNA stability in 786-O an A498 cells with the indicated treatments as determined by RT-qPCR. Actinomycin D was used to block the transcription of PDGFRβ. Data are presented as mean±SD. *p<0.05, ***p<0.001, ns, not significant, by Student's t test (B) or 1-way ANOVA (D, E)

Supplementary Methods

Plasmid constructions and siRNA interference

For VHL and PDGFRβ overexpressing plasmids, cDNAs were synthesized and cloned into pLVX vector (Guangzhou Bioyard Biotechnology Development Co., Ltd). The VHL or PDGFRβ overexpression plasmids were co-transfected into HEK293T cells with the lentivirus packaging plasmids using Lipofectamine 3000 (Invitrogen, CA, USA). After 48 h transfection, the supernatants containing lentivirus were collected to infect tumor cells, and stably transfected cells were selected with puromycin. Specific siRNAs targeting VHL, HIF-1α, HIF-2α, LDHA, LDHB and PDGFRβ, and scrambled control siRNA were transfected into the indicated cells with Lipofectamine RNAiMAX (Invitrogen, CA, USA) according to the manufacturer's recommendations.

siRNA (target sequence)	
siHIF-1α	GTGATGAAAGAATTACCGAAT
siHIF-2α	GCCGTACTGTCAACCTCAA
siLDHA	CCACCATGATTAAGGGTCTTT
siLDHB	GCTTATTTCTTCAGACACCTA
siPDGFRβ	CCAAAGGAGGACCCATCTATA
siVHL	TATCACACTGCCAGTGTATAC

Western blot

Total protein was extracted using RIPA lysis buffer, and the protein concentration was measured with PierceTM BCA protein assay kit (Thermo, USA). Equal amounts of proteins were separated by SDS-PAGE gel and transferred to PVDF membrane (Merck Millipore, Billerica, MA, USA). After blocked with 5% milk, the membranes were then incubated with the following primary antibodies overnight at 4 °C: pan anti-Kla (PTM Bio Inc, 1:1000), anti-H3K18la (PTM Bio Inc, 1:1000), anti-Histone-H3 (Abcam, 1:1000), anti-VHL (Abcam, 1:1000), anti-HIF1α (Abcam, 1:1000), anti-HIF2α (Abcam, 1:1000), anti-β-Tubulin (Abcam, 1:5000), anti-Glut1 (Abcam, 1:1000), anti-EP300 (Abcam, 1:1000), anti-PDGFRβ (Abcam, 1:1000), anti-LDHA (CST, 1:1000), anti-c-Myc (CST, 1:1000) and anti-LDHB (Millipore Sigma, 1:1000). Then membranes were incubated with HRP-conjugated secondary antibodies, and detected

using Chemiluminescence instrument (Bio-Rad, USA).

Immunohistochemistry staining

Human Paraffin-embedded ccRCC and normal kidney samples were deparaffinized and rehydrated. Antigen retrieval was performed using 0.01M citrate buffer (pH 6.0) in a pressure cooker and non-specific binding was blocked with 5% BSA. Anti-L-Lactyl Lysine Rabbit mAb (PTM Bio Inc, 1:100), anti-Lactyl-Histone H3 (Lys18) Rabbit mAb (PTM Bio Inc, 1:100) and anti-PDGFRβ (Abcam, 1:100) were added on the sections and incubated overnight at 4°C. HRP-conjugated secondary antibody and DAB staining were performed using an anti-mouse/rabbit IHC Secondary Antibody Kit (ZSGB-BIO) according to the manufacturer's instructions.

CCK-8 assay

For CCK-8 cell proliferation assay, 500 cells were seeded in 96-well plates with fresh medium. At each time point, 10 μl CCK8 (Dojindo, Japan) solution was added to each well and incubated at 37 °C for 1 h. The absorbance was measured at 450 nm using a spectrophotometric plate reader (BioTek ELX800, USA) at given gradient time (1, 2, 3 and 4 day).

Transwell assay

For the transwell migration assay, 30,000 cells resuspended in serum-free medium were seeded onto the upper chamber of 24-well transwell chamber (Corning Falcon, NY, USA), with the lower chamber filled with medium supplemented with 10% FBS as the chemo-attractant. The migrated cells were fixed, stained, and counted under a light microscope.

Chromatin immunoprecipitation (ChIP) assay

The ChIP assay was conducted using the Pierce™ Magnetic ChIP Kit (Thermo Fisher Scientific) according to the manufacturers' instructions. Briefly, after cross-linking and chromatin digestion, digested chromatin was incubated with 5 μg of anti-H3K18la or anti-EP300 or anti-H3K27ac (CST), and anti-IgG antibodies at 4 °C

overnight, respectively. Then, protein A/G magnetic beads were added and incubated for another 4 h. The immunoprecipitated DNA was purified and then used to construct ChIP-seq libraries or analyzed by qPCR. The primers used to analyze the enriched DNA are listed as follows.

Primers	
PDGFRβ-F (ChIP)	AGGCCACTCTTTGACAAAA
PDGFRβ-R (ChIP)	TGTTTTCAATTTCAGTTTTTT

RNA isolation and quantitative real-time PCR (qRT-PCR)

Total RNA was extracted using TRIzol reagent (Invitrogen, CA, USA). Isolated RNA was used for reverse transcription with the HiScript II 1st Strand cDNA Synthesis Kit (Vazyme, Nanjing, China) according to the manufacturer’s instructions. The cDNA was used for qPCR using the HiScript II One Step qRT-PCR SYBR Green Kit (Vazyme, Nanjing, China) on a CFX96 real-time PCR detection system (Bio-Rad). β-actin was used as an internal control for calculating relative mRNA expression. The qPCR primers were listed as follows.

Primers	
PDGFRβ-F (qPCR)	AGCACCTTCGTTCTGACCTG
PDGFRβ-R (qPCR)	TATTCTCCCGTGTCTAGCCCA
β-Actin-F (qPCR)	CATGTACGTTGCTATCCAGGC
β-Actin-R (qPCR)	CTCCTTAATGTCACGCACGAT

Seahorse assay

To determine the extracellular acidification rate (ECAR), a XF24 Extracellular Flux Analyzer (Seahorse Bioscience) was used. Briefly, 2×10^4 786-O cells with indicated treatments were plated in assay medium (XF Base Medium containing 2 mM L-glutamine) under basal conditions and treated with 10 mM glucose, 1 μM Oligomycin (Seahorse Bioscience), and 50 mM 2-deoxy-D-glucose (2-DG) (Sigma-Aldrich) according to the manufacturer's instructions. Data were normalized by the protein quantification.

Flow cytometry

Cells were collected, washed and resuspended in PBS. For measurement of PDGFRβ expression, cells were stained with anti-PDGFRβ or isotype-control antibodies for half an hour. Then, cells were washed three times with PBS and stained with Alexa Fluor 488-conjugated goat anti-mouse IgG (Invitrogen) for another 20 min. Samples were analyzed with a Beckman CytoFLEX Flow cytometer and mean fluorescence intensity (MFI) was used to detect the expression of PDGFRβ. For apoptosis analysis, cells were collected and incubated with Annexin V-APC (Multi Science, China) in binding buffer for 5 min at room temperature, dyed with PI solution, and then immediately analyzed by CytoFLEX Flow cytometer. FlowJo10 software was used to analyze the data.

Lactate measurement

Lactate levels were measured using a Lactate Colorimetric/Fluorometric Assay Kit (Biovision) according to the manufacturer's instructions. Briefly, cell pellet or tumor tissue was lysed in the buffer provided by the kit and centrifuged to collect the supernatant. The supernatant was then mixed with the assay solution. The absorbance was measured at 570 nm using a spectrophotometric plate reader (BioTek ELX800, USA) and the readout was normalized by the protein amounts determined by PierceTM BCA protein assay kit (Thermo, MA, USA).

Glucose concentration measurement

The glucose concentration was determined by the Glucose Colorimetric Kit (Sigma) according to the manufacturer's instructions. Briefly, cell supernatant was mixed with the assay solution. The absorbance was measured at 570 nm using a spectrophotometric plate reader and the readout was normalized to the cell number.

PDGFβ stimulation assay

ccRCC cells were seeded into 6-well plates one day before experiments. On the next day, cells were rinsed twice with PBS and cultured in medium without FBS for 24 hours. Then, serum-starved cells were stimulated with medium containing 10 ng/ml

PDGF-BB (R&D Systems). After PDGFβ stimulation, cells were prepared for further experiments.

Uncropped versions of western blot images

Full uncropped blots for Figure 1F

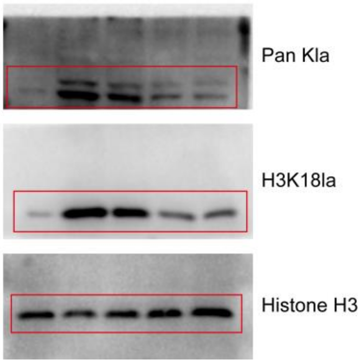

Full uncropped blots for Figure 1G

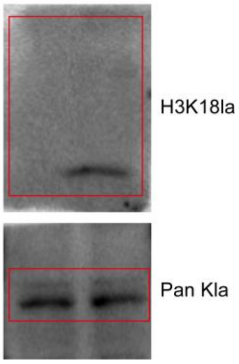

Full uncropped blots for Figure 1K

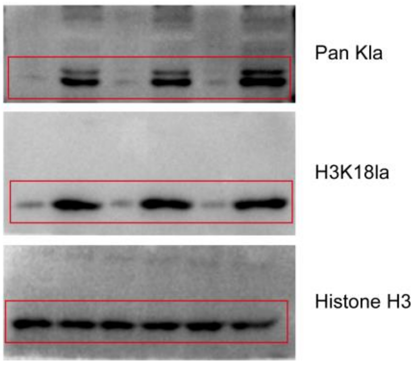

Full uncropped blots for Figure 2A

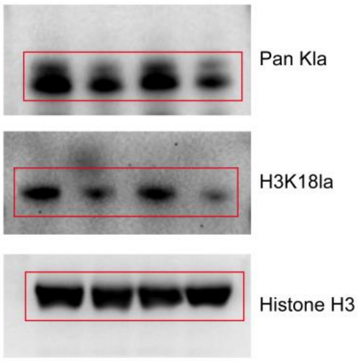

Full uncropped blots for Figure 2B

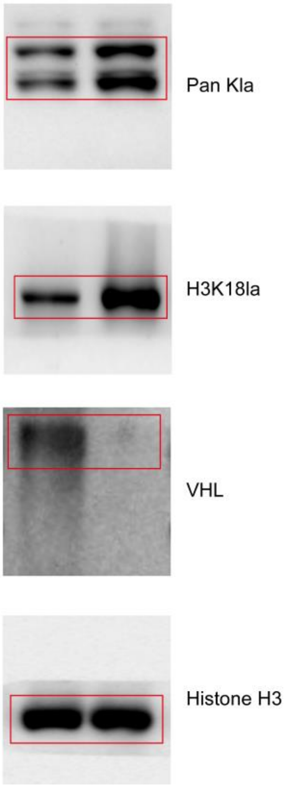

Full uncropped blots for Figure 2C

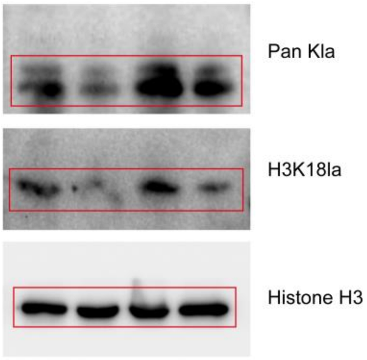

Full uncropped blots for Figure 3B

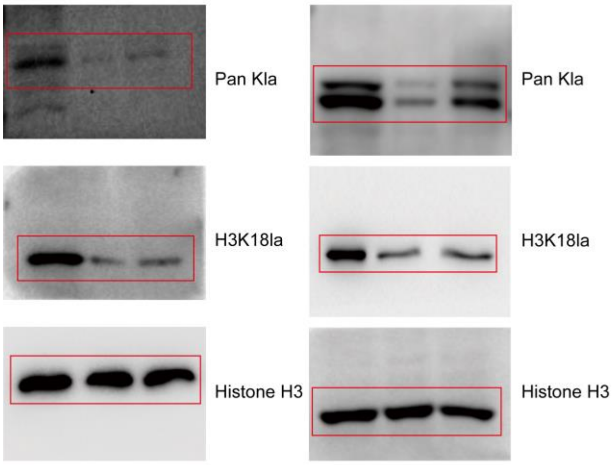

Full uncropped blots for Figure 3E

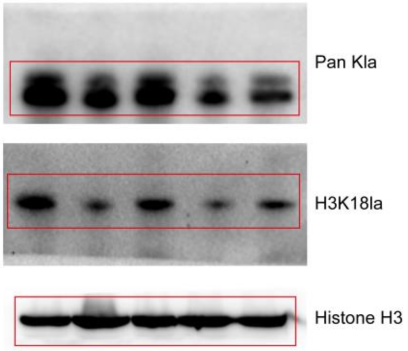

Full uncropped blots for Figure 3D

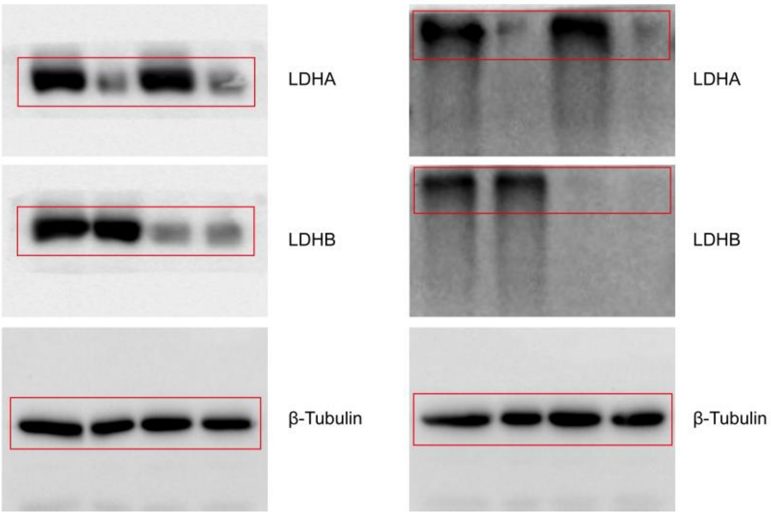

Full uncropped blots for Figure 4I

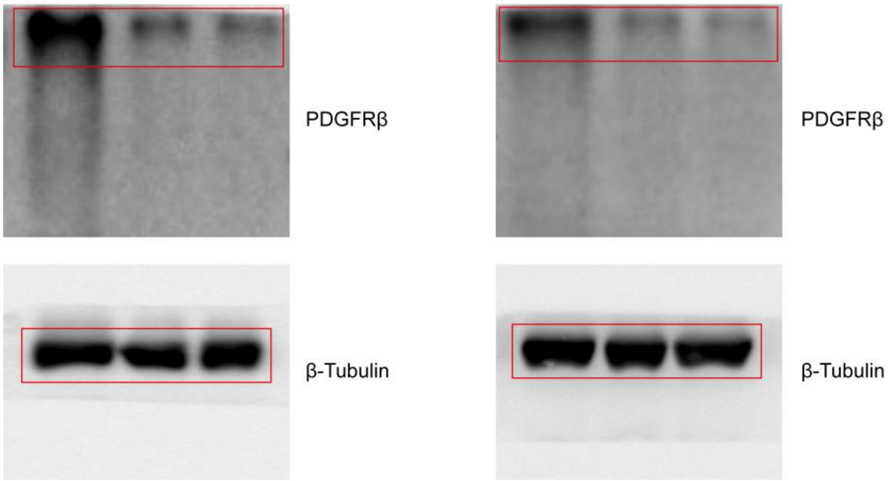

Full uncropped blots for Figure 6C

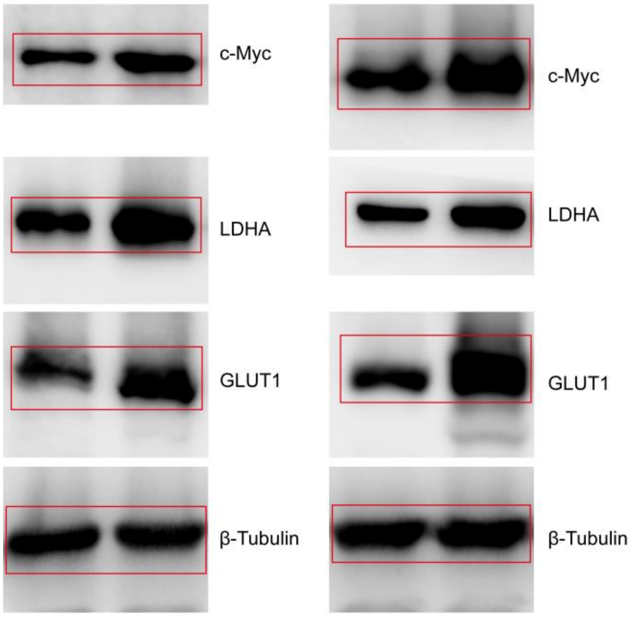

Full uncropped blots for Figure 6J

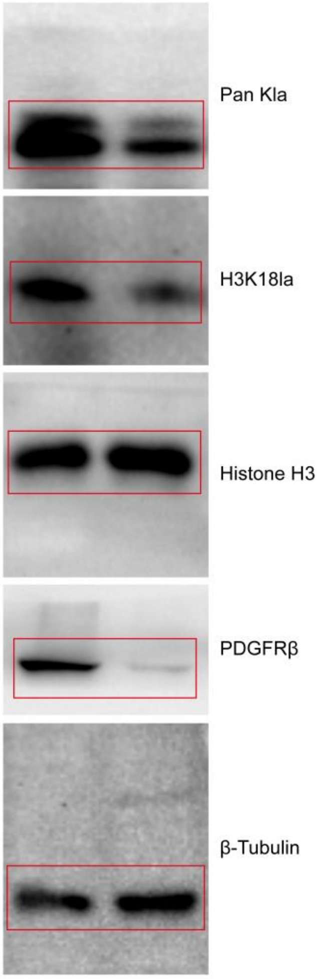

Full uncropped blots for Figure 6E

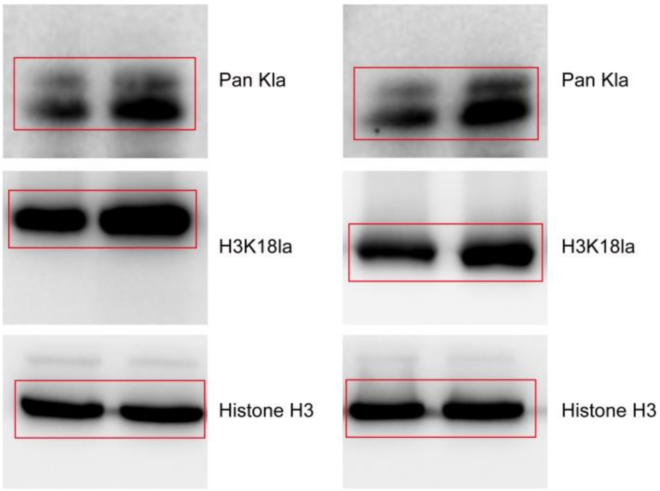

Full uncropped blots for Figure 6M

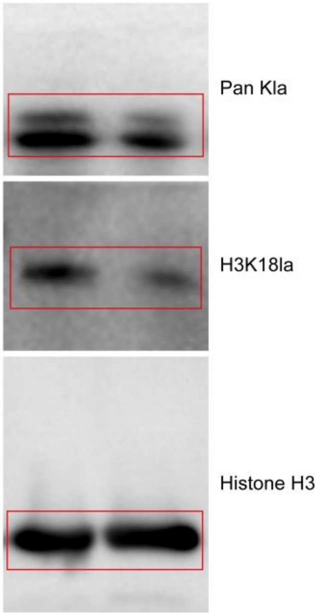

Full uncropped blots for Figure S2A

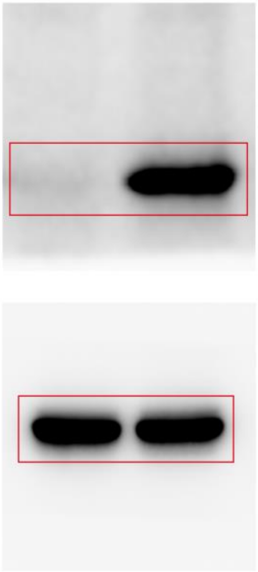

Full uncropped blots for Figure S2B

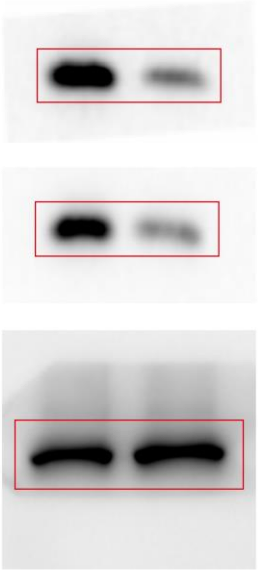

Full uncropped blots for Figure S4C

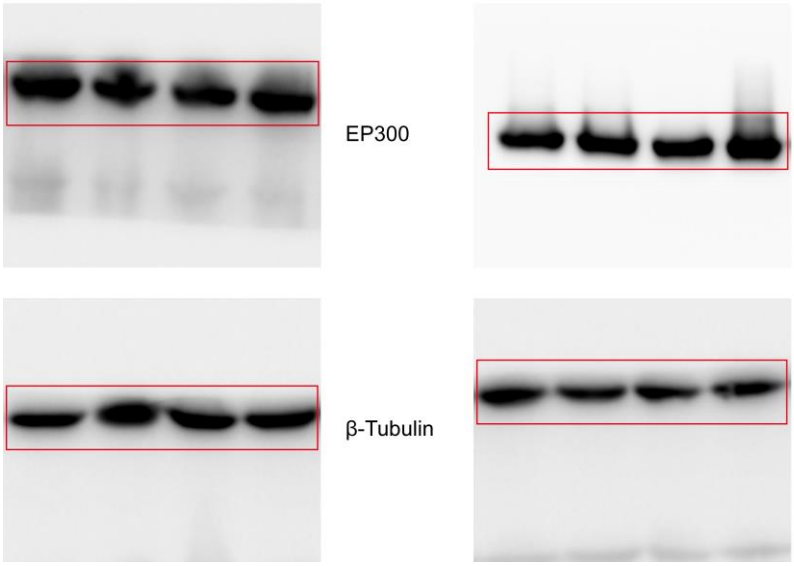

Full view of lung micro-metastasis

Full view of lung micro-metastasis for Figure 7J

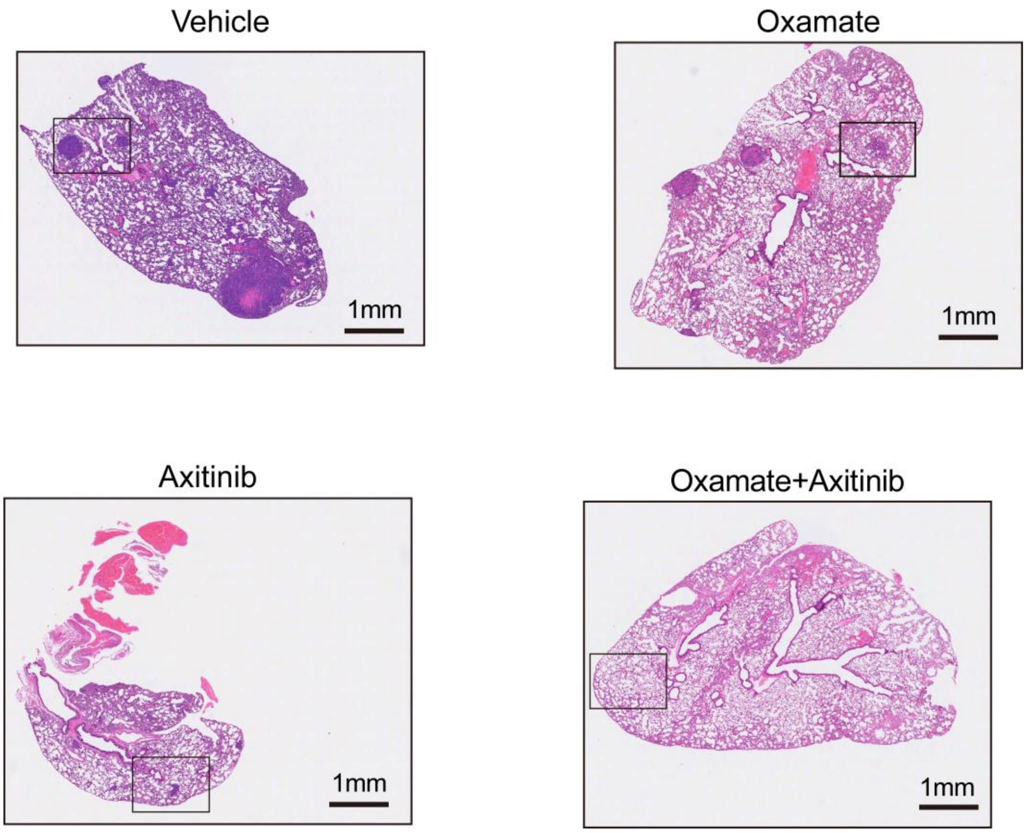

Supplement: Supplementary file 1 — Supplementary figures and methods. [file ijbsv18p3470s1.pdf]
